# 

**Published:** 1887-03

**Authors:** 


### CONTENTS:

ARTICLE I.—Odonto-Chirurgical Society........ ............. ................... 481

" II.—Dentistry a Specialty of Medicine. By B. Merrill Hopkinson, D. D. S., M. D.,............................................ 499

" III.—Mirror Topped Syphon Tongue Holder. Invented by Dr. D. Genese.................... ......................................... 506

" IV.—Dental Jurisprudence. By Richard Grady, D. D. S........ 508

" V.—International Medical Congress.............. ..................... ... 513

### EDITORIAL, ETC.

University of Maryland, Dental Department—Annual Commencement for 1887.......... ................................................... 520

The First Colored Dental School................. .................................... 523

International Medical Congress.............................................. ...... 525

### OBITUARY.

Dr. John G. Wayt .............. ................ ....................................... 525

### MONTHLY SUMMARY.

Chloral Hydrate as a Vesicant........... ............................................... 527

A Dear Kiss.... ............ ........... ......................... ..... .................. 528

International Medical Congress .................................................... 528

